# Mimicking focused ultrasound with a loop coil in acoustic radiation force imaging

**DOI:** 10.1002/mrm.70014

**Published:** 2025-08-24

**Authors:** Kristen Zarcone, Anuj Sharma, William A. Grissom

**Affiliations:** ^1^ Department of Biomedical Engineering Case Western Reserve University Cleveland Ohio USA

**Keywords:** MR‐ARFI, focused‐ultrasound

## Abstract

**Purpose:**

To enable development of MR‐acoustic radiation force imaging (MR‐ARFI) methods for targeting ultrasound in human subjects without the regulatory, acoustic, or hardware challenges associated with actual transcranial ultrasound setups.

**Methods:**

MR‐ARFI is a phase‐contrast imaging method that measures focal tissue displacement produced by an ultrasound transducer, when the transducer is pulsed simultaneously with a motion encoding gradient. The ultrasound‐induced focal phase shift can be mimicked with a small loop coil that is driven by a DC pulse to produce a resonance frequency offset at the same time as the ultrasound pulse in an MR‐ARFI pulse sequence. A coil was designed and built for use in MR‐ARFI. Its focus size was characterized, its field map was measured, and volunteer experiments were performed to demonstrate its function in transcranial phase‐contrast and magnetization‐prepared MR‐ARFI.

**Results:**

Off‐resonance field maps measured with the constructed loop coil were within 0.87% of simulations in a slice 15 mm from the coil's surface. Its “focus” further had a full‐width‐at‐half‐maximum of 22.9 mm in simulation versus 22.7 mm in the field map. In vivo results showed that the same coil driven with 13.7 mA current produced a phase shift corresponding to a realistic effective displacement of 3.5 μm in a slice 19 mm from the coil in MR‐ARFI.

**Conclusion:**

A pulsed DC loop coil can mimic ARF‐induced displacements in vivo, facilitating development of MR‐ARFI methods in vivo.

## 1 INTRODUCTION

Transcranial ultrasound stimulation (TUS) is developing as a powerful modality for noninvasive neuromodulation with the potential to treat numerous conditions such as Parkinsonian tremor, chronic pain, substance use disorder, and more.^1^, ^2^, ^3^, ^4^ However, the skull attenuates and distorts the ultrasound focus, making precise targeting of neural circuits difficult. MR‐acoustic radiation force imaging (MR‐ARFI) can be used to target TUS and other ultrasound therapies without heating.^5^, ^6^, ^7^ MR‐ARFI leverages the acoustic radiation force (ARF), which is a transfer of momentum from ultrasound waves to tissue, and which causes bulk tissue displacement on the order of microns. By applying a motion‐encoding gradient pulse (MEG) simultaneously with the ultrasound pulse, MR‐ARFI sequences encode the tissue displacement in the phase of transverse magnetization, and the resulting imaged phase shift is proportional to the average displacement that occurred during the MEG.

As the only nonthermal, noninvasive method that can visualize an ultrasound focus in the brain, considerable effort has been invested in developing MR‐ARFI pulse sequences, reconstructions, and processing, with the goals of maximizing its sensitivity, minimizing ultrasound exposure, minimizing scan time, and minimizing its sensitivity to physiological phase artifacts.^9^, ^10^, ^11^, ^12^, ^13^, ^14^, ^15^, ^16^ While phantom experiments are sufficient for preliminary MR‐ARFI pulse sequence development, testing pulse sequences in human subjects is necessary to capture real‐world effects such as phase artifacts induced by physiological motion, in order to create sequences robust to those artifacts. However, in vivo MR‐ARFI development is impeded by three factors. First, MR‐ARFI protocols may come close to or exceed current US Food and Drug Administration (FDA) ultrasound intensity or mechanical index limits,^17^ making institutional review board (IRB) approval for in vivo MR‐ARFI development challenging. For example, recent preclinical MR‐ARFI work used focal pressures (assuming 39% skull transmission) corresponding to mechanical indices of 2.7‐4.3, which are above the FDA's limit of 1.9.^18^ Guidelines for safe ultrasound exposure in TUS have been generated by an expert panel^17^ but have not yet been adopted by the FDA.

However, even with IRB approval for TUS studies, reliable and repeatable transducer positioning and acoustic coupling are challenges that are being addressed in parallel development work,^18^, ^19^ but it is presently difficult to know whether weak focal displacement or a complete lack of focal displacement are the result of an acoustic failure, skull attenuation which varies widely between subjects, or a failure of the MR‐ARFI acquisition. Finally, MRI‐compatible TUS transducers and generators cost up to several hundred thousand dollars, are not yet widely available, and most lack FDA approval and therefore require institutional investigational device reviews at each institution, the procedures and outcomes from which can vary widely between institutions. Even when available at a site, they must often be shared between bench development work, out‐of‐scanner TUS experiments and treatments, and imaging development. They also require considerable time to set up in preparation for an experiment. In combination, these factors have slowed the development of MR‐ARFI methods in vivo.

In this work, we describe how a loop coil driven by a pulsed DC source (in the present work, an arbitrary waveform generator that is readily available in most laboratories) can produce a focal phase shift mimicking that produced by ultrasound‐induced tissue displacement, to enable safe and repeatable MR‐ARFI development in vivo. The full‐width‐at‐half‐maximum of the focus produced by such a coil was characterized in simulation as a function of coil diameter and depth. A coil was built and field mapping was used to validate the field it produced versus simulations. It was then deployed in transcranial MR‐ARFI scans in a healthy volunteer.

## 2 METHODS

**FIGURE 1:**
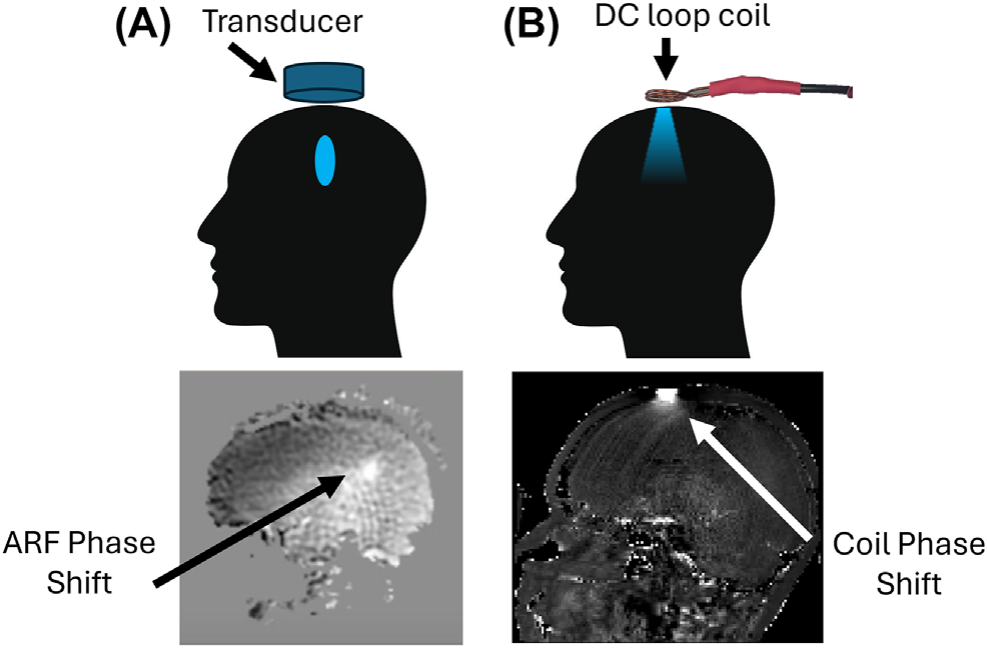
(A) (Top) An ultrasound transducer acoustically coupled at the top of the head will generate a focused pressure field in the brain (blue oval) and (Bottom; adapted from Ref. [8]). ARF‐induced tissue displacement at the focus generates a detectable phase shift in an MR‐ARFI image. (B) (Top) A DC loop coil placed on top of the head (Bottom) creates a localized phase shift that mimics the ultrasound focus in an MR‐ARFI sequence.

Figure 1 illustrates the concept of the DC loop coil for MR‐ARFI. Figure 1A shows an ultrasound transducer placed on a subject's head, and a displacement‐induced MR‐ARFI phase shift map measured in a subject's brain (adapted from Ref. [8]), which is focal with a full‐width‐at‐half‐maximum of around 2 centimeters. As shown in Figure 1B, in place of the ultrasound transducer, we propose to place a small (up to a few centimeters in diameter) loop coil above the subject's head and aligned with $B_{0}$. This coil generates a small focal $B_{z}$ field that, when switched on at the same time that the ultrasound would be pulsed in an MR‐ARFI sequence, produces a focal phase shift in an MR‐ARFI image.

**FIGURE 2:**
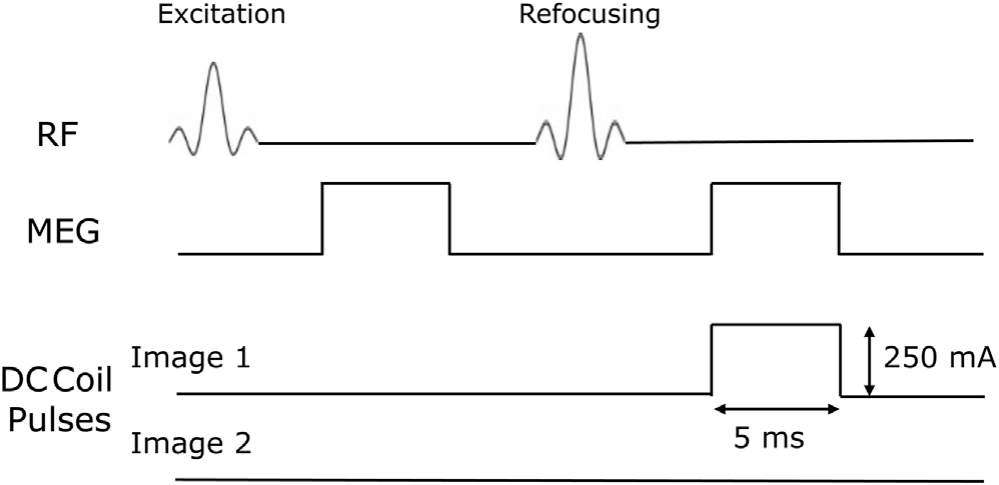
The coil pulsing scheme used to incorporate a DC loop coil in a spin‐echo MR‐ARFI pulse sequence. The sequence acquires two images, and in one image the coil is triggered by the scanner to pulse simultaneously with a motion encoding gradient.

Figure 2 illustrates a scheme to incorporate a DC loop coil in a typical MR‐ARFI pulse sequence, which includes MEGs placed on either side of a refocusing pulse.^5^ To remove background phase, conventional phase‐contrast MR‐ARFI collects at least two images with opposite tissue displacement phase, or with zero phase (no ultrasound pulse) in one image. The coil's pulse generator is triggered simultaneously with an MEG, and two images are collected. The phase difference between the images is then proportional to displacement. The pulsing scheme triggers the pulse generator with one of the MEGs in one image and collects a second image without a pulse. Alternative pulsing schemes are shown in Supporting Information Figure S1.

### 2.1 Coil simulations

To calculate the field shifts produced by a DC loop coil as a function of depth and coil diameter, the Biot–Savart law was numerically solved for loops of current‐carrying wire in Matlab 2024a (Mathworks, Natick, MA, USA) using elliptic integrals. In all simulations, the current in the coil was set to 250 mA. These calculations were first used to measure the FWHM's of the generated $B_{z}$ fields as a function of coil diameter and the distance of an imaging slice from the surface of the coil. To do this, $B_{z}$ was calculated over a centered 1D line parallel to the surface of the coil, comprising 1500 grid points with a 400 mm range centered at the coil. The calculation was performed 1201 times at slice distances ranging from 5 mm to 70 mm away from the surface of the coil and for a range of coil diameters from 5 mm to 50 mm. Then, to validate the constructed coil described below, $B_{z}$ was calculated over a 3D grid for a loop coil with 2 turns and a 20 mm diameter. The matrix consisted of 128 × 128 × 1230 points with a FOV of 17.5 × 17.5 × 6.0 cm^3^. $B_{z}$ values were then averaged along the z axis in order to match the 2 mm slice thickness of measured $B_{z}$ field maps, resulting in a 3D array of 128 × 128 × 30 points. The z dimension was sampled more finely than the x and y dimensions since the field varied much more rapidly in z.

### 2.2 Coil construction

A DC loop coil with 2 turns and a diameter of 20 mm was constructed for phantom validation and in vivo MR‐ARFI using 14 AWG copper magnet wire. A two‐turn coil was chosen because its simulated $B_{z}$ field generated an expected phase shift of similar magnitude as ARF displacement (between 0.5–6 μm) with low current (13.7 mA). The coil was soldered to a long BNC cable, and heat shrink wrap was applied to the exposed wire. The cable was connected to a Mini‐Cicuits BLP‐15+ (Mini‐Circuits, Brooklyn, NY, USA), low‐pass filter with a 17 MHz corner frequency to mitigate noise and electromagnetic interference, and connected to the function generator in the control room as described below. The coil was safety tested for interactions with the RF and gradient systems prior to use on human subjects by verifying a lack of arcing and detectable temperature rise with an infrared thermometer in the MRI scanner. International Electrotechnical Commission (IEC) guidelines require the temperature of any device held in prolonged contact with the skin remain below $43^{\circ }$ C.^20^ To further verify that the coil operates within this requirement, its temperature was measured on the bench with an infrared thermometer over a three minute period while supplied with seven constant current levels: 250 mA, 500 mA, 1.0 A, and 2.0 A, 4.0 A, 8.0 A, and 12.0 A. No temperature rise was measured from 250 mA to 4.0 A. After three minutes, a $0.7^{\circ }$ C rise was measured at 8.0 A, and a $1.5^{\circ }$ C rise was measured at 12.0 A. Even if the coil started at a body temperature of $37^{\circ }$ C, these temperatures would not violate the IEC requirement. The maximum simulated $B_{z}$ value produced by the coil at 250 mA was 41.2 µT, which is well below the FDA's 8 T limit. The resistance of the combined coil, cable, and low‐pass filter were measured at 1.6 Ω. A voltage of 450 mV was supplied from the function generator, which produced a current of 13.7 mA in the coil in in vivo MR‐ARFI experiments.

### 2.3 Phantom experiments

A gradient‐recalled echo (GRE) $B_{0}$ map was acquired on a 3T MRI scanner (Vida, Siemens Healthineers, Erlangen, Germany) to measure the $B_{z}$ field generated by the constructed loop coil. The sequence used TEs of 4.52 ms and 6.98 ms, a TR of 1000 ms, a pixel bandwidth of 260 Hz/pixel, and it acquired a 128 × 128 image matrix with a 17.5 × 17.5 cm^2^ FOV, and 30 slices with 2 mm thickness. The loop coil was affixed to a spherical ball phantom using tape. The phantom and coil were placed in the scanner such that the coil's $B_{z}$ field was aligned with $B_{0}$, and imaged using a twenty‐channel head coil. A current of 250 mA was supplied to the coil via a Tenma 706610 DC power supply (Tenma Corporation, Tokyo, Japan) for the entire scan duration, switched on after pre‐scan and shimming.

### 2.4 In vivo experiments

With IRB approval, the DC loop coil was affixed to the top of a healthy male volunteer's head using Coban wrap, and MR‐ARFI images were acquired. The coil was positioned approximately two centimeters from the volunteer's midline so that the focal phase shift appeared in the cortex rather than over the central sulcus. The volunteer's head was then placed in the coil with a slight tilt so that the coil's $B_{z}$ field remained aligned with $B_{0}$. Images were acquired with both a conventional phase‐contrast MR‐ARFI pulse sequence (“phase‐ARFI”), and a magnetization‐prepared magnitude‐based MR‐ARFI (“mag‐ARFI”) pulse sequence,^10^, ^21^ both using the pulsing scheme of Figure 2. The coil was pulsed synchronously with the sequences' second MEG using an Agilent 33220A arbitrary waveform generator (Agilent Technologies, Santa Clara, CA, USA) that was triggered by the scanner to generate a 5 ms‐long pulse, with the voltage set to 450 mV to produce 13.7 mA in the coil. The phase‐ARFI sequence used a 5 ms MEG duration and 40 mT/m amplitude, a TE of 25 ms, and TR of 500 ms. It acquired a 128 × 128 image matrix with a 23.0 × 23.0 cm^2^ FOV and ten slices with 4.0 mm thickness. The pixel bandwidth was 260 Hz/pixel, and maps were acquired in both the axial and sagittal orientations. The effective tissue displacement produced by the loop was calculated using the MEG parameters as though the pulse were applied to both images, using^5^:

(1) $$\Delta_{x}=\frac{\Delta_{\phi }}{2\gamma \tau G},$$

where $\Delta_{x}$ is the effective displacement, $\Delta_{\phi }$ is the phase difference between the two images, γ is the gyromagnetic ratio, τ is the duration of the MEG (5 ms), and G is the MEG amplitude (40 mT/m).

The mag‐ARFI sequence^10^, ^21^ was a magnetization‐prepared turbo spin echo acquisition that began each TR with a spin‐echo magnetization preparation stage with MEGs and the DC loop coil pulse following the Image 1 scheme in Figure 2 with the same MEG amplitudes and durations as the phase‐ARFI scan. Image 2 is not acquired in mag‐ARFI since a single image directly shows the focus, without subtraction. To obtain a bright signal at the focus, the tip‐up pulse had a 90‐degree phase shift relative to the excitation pulse. The mag‐ARFI sequence's TR was 6000 ms, its TE was 90 ms, its pixel bandwidth was 222 Hz/pixel, and its matrix size and volume coverage were the same as the phase‐ARFI sequence.

## 3 RESULTS

### 3.1 Full‐width‐at‐half‐maximum simulation

**FIGURE 3:**
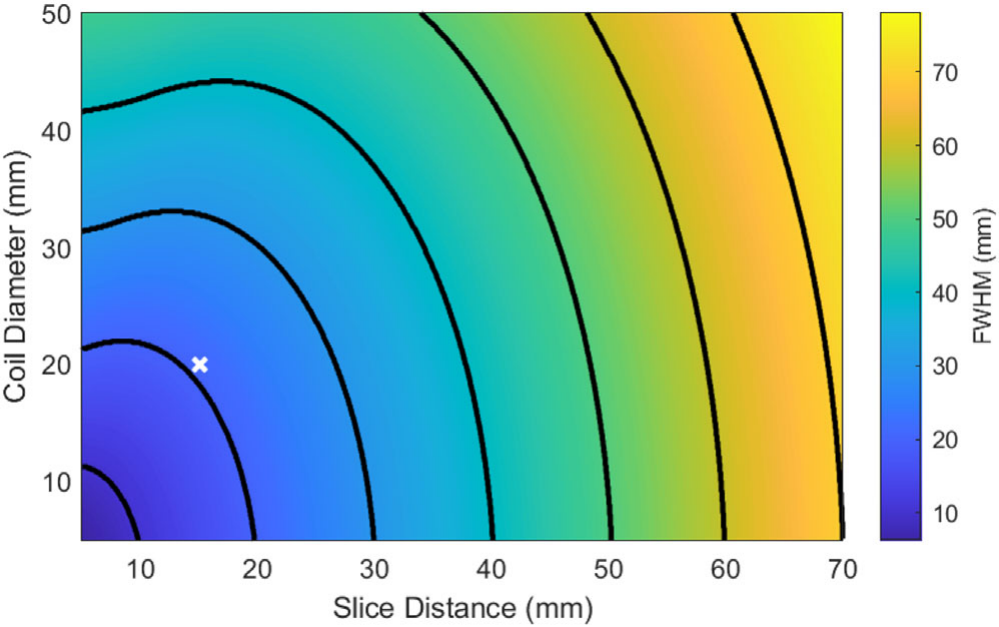
The FWHM of the $B_{z}$ focus generated by a current‐carrying loop as a function of both coil diameter and transverse slice distance from the coil. Calculations were made over a range of coil diameters from 5 to 50 mm and transverse slice distance 5 to 70 mm away from the surface of the DC loop. Isocontours are shown at 10 mm FWHM intervals, where the left‐most contour represents a 10 mm FWHM, and the right‐most contour represents a 70 mm FWHM. A white “x” indicates the 20.5 mm FWHM of the 2‐turn, 20 mm‐diameter loop at a slice distance of 15 mm from the coil's surface.

Figure 3 shows how the FWHM of the $B_{z}$ field measured in a slice parallel to a loop coil depends on the coil's radius and distance of the slice from the coil. Combinations of coil radius and slice distance that result in constant FWHM are depicted with isocontours. Tracing each contour from left to right along the slice distance axis, the teardrop shape of the coil's dipole field is reflected in the upward slope of each contour, until it rapidly drops off starting at a slice distance of approximately 2/3 the coil diameter. A FWHM of 20 mm can be achieved with a coil diameter of 20 mm, 13.3 mm from the coil as well as with a 10 mm diameter, 19 mm from the coil. A larger FWHM of 30 mm can be achieved with a coil diameter of 30 mm and a slice position 19 mm from the coil, as well as with a coil diameter of 10 mm, and 29 mm from the surface of the coil.

### 3.2 Field mapping

**FIGURE 4:**
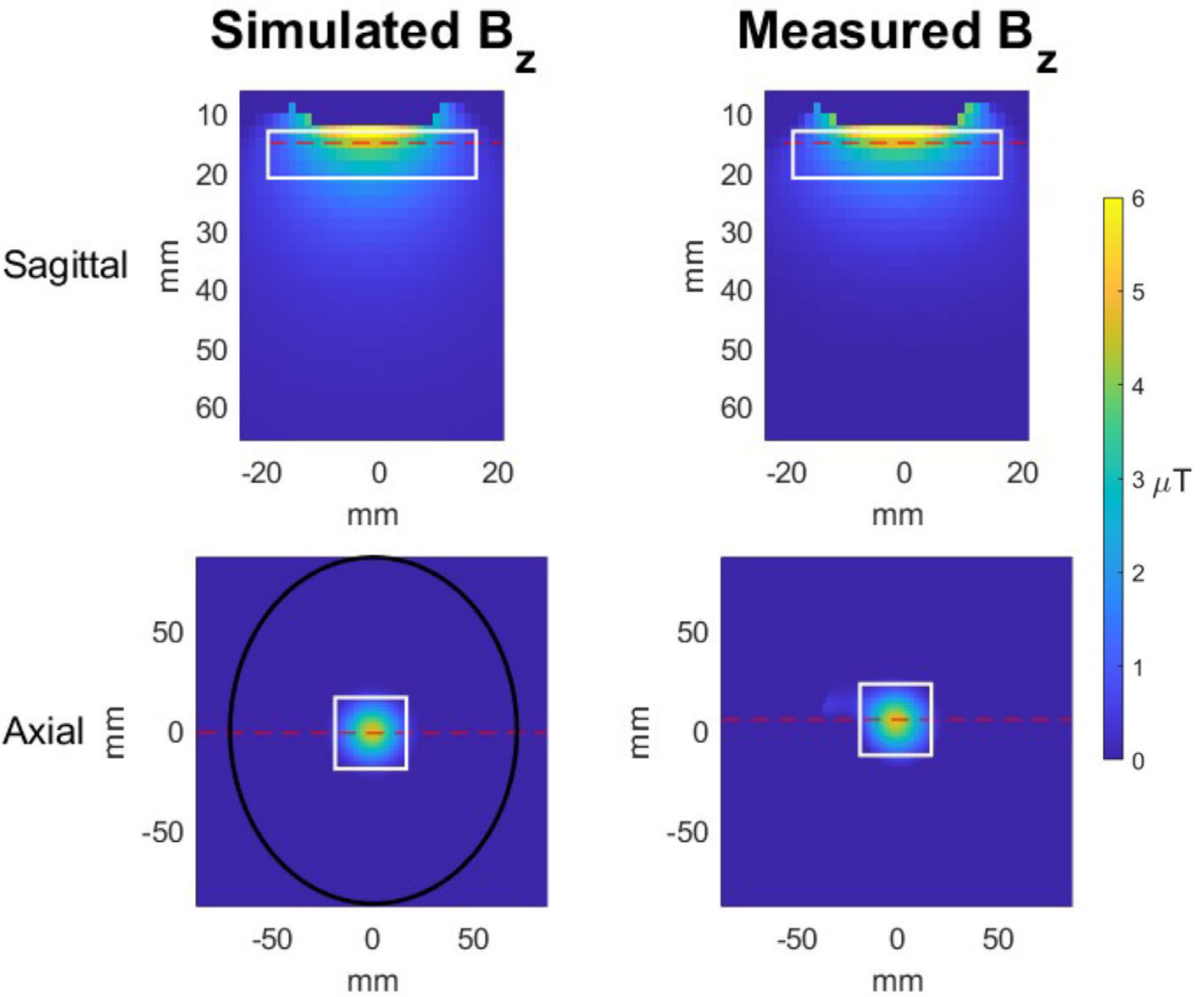
Sagittal (top) and axial (bottom) planes of the simulated (left) and measured (right) $B_{z}$ maps. Red dashed lines show the locations of the corresponding axial and sagittal slices in the other plane; the sagittal plane was centered in the axial plane, and the axial plane was positioned 15 mm from the loop coil. For illustration, the simulated axial $B_{z}$ map contains a human head‐sized ellipse with a major diameter of 173 mm and minor diameter of 143 mm (bottom left).

Figure 4 shows sagittal and axial slices of the simulated and measured $B_{z}$ maps for the constructed 2‐turn, 20 mm‐diameter coil; Supporting Information Figures S2–S5 show additional slices to further illustrate the 3D spatial variation of $B_{z}$. The measured sagittal field map shows signal dropout due to dephasing and phase wrapping very close to the coil. A mask was applied to the simulated $B_{z}$ map to mimic the signal dropout in the acquired map. The loop's simulated $B_{z}$ map had a maximum value of 4.62 μT and a 22.9 mm FWHM in a slice 15 mm from the coil. This value is wider than the 20.5 mm FWHM shown in Figure 3, due to averaging over the 2 mm slice width to match the experiment. In close agreement, the maximum value of the measured $B_{z}$ map was 4.74 μT with a 22.7 mm FWHM in a slice 15 mm from the coil.

Mean and standard deviation field values were determined over a 35.5 × 35.5 × 8 mm^3^ volume 13 mm from the coil's surface and centered on the coil, depicted by a white box on the sagittal images. For the simulated maps, the mean over this volume was 1.40 μT, with standard deviation of 1.14 μT. Similarly, the mean over this volume in the field maps was 1.27 μT, with a 1.33 μT standard deviation. The mean value over a 35.5 × 35.5 mm^2^ area in the axial slices depicted by a white box shown in Figure 4 15 mm from the surface of the coil for both simulation and measured field maps was calculated. The mean in the simulated $B_{z}$ map was 1.59 μT with a standard deviation of 1.25 μT, while the the mean over the measured $B_{z}$ area was 1.68 μT with a standard deviation of 1.30 μT.

### 3.3 In vivo results

**FIGURE 5:**
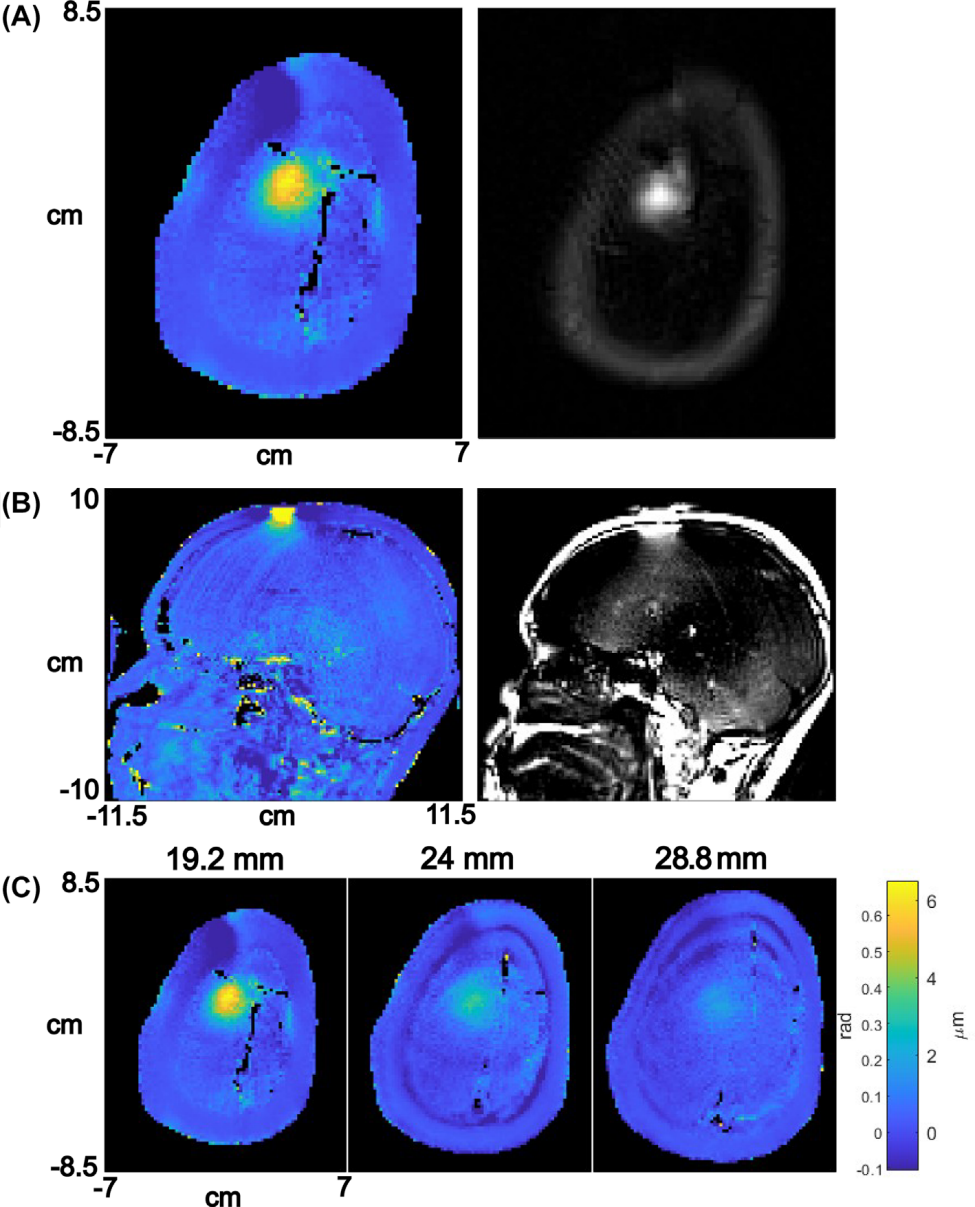
(A) In vivo phase‐ARFI map (left) and Mag‐ARFI image (right) generated with the loop coil in an axial slice. (B) A phase‐ARFI map and a mag‐ARFI image in a sagittal slice. (C) Phase‐ARFI maps acquired at three distances from the loop coil/depths into the brain. The colorbar in (C) applies to all the phase‐ARFI maps. The colormap's phase shift range corresponds to a frequency shift range of −3.4 to 22.1 Hz.

Figure 5A shows an axial phase‐ARFI map and a mag‐ARFI image in the same slice. The FWHM of the phase‐ARFI map was 18 mm in the L/R direction and 19.8 mm in the A/P direction. The FWHM of the Mag‐ARFI images was slightly smaller at 14.4 mm in both the L/R and A/P directions. Figure 5B further shows a map and image in a sagittal plane. Figure 5C illustrates the fall‐off of the effective displacement with distance from the coil: the slice at 19.2 mm had a maximum displacement of 6.62 μm, a slice at 24 mm had a maximum displacement of 3.46 μm, and a slice at 28.8 mm had a maximum displacement of 1.92 μm. Using an MR‐ARFI sequence with the same imaging parameters used in vivo, phantom maps acquired using the DC loop coil to induce an ARF‐mimicking phase shift showed agreement to simulations within 0.1 radians across all slices (results shown in Supporting Information Figure S6), corresponding to an effective displacement agreement within 0.93 µm. This confirms controllability of the effective displacement applied by the coil.

## 4 DISCUSSION

We have demonstrated that DC loop coils can mimic the effect of ultrasound in producing focal, tissue displacement‐like phase shifts in MR‐ARFI scans. This offers a simple, repeatable, and low‐cost setup for MR‐ARFI development in human volunteers, enabling advances in MR‐ARFI acquisitions to be made with minimal regulatory hurdles and experimental difficulties. Testing MR‐ARFI pulse sequences in human subjects is important to evaluate them in the presence of real‐world effects such as physiological phase artifacts that can obscure an acoustic focus. For example, the coils could be used to empirically optimize MR‐ARFI pulse sequence parameters to maximize displacement signal‐to‐noise ratio, while avoiding the possibility of excessive ultrasound exposure and maximizing repeatability of the measurements. Coil selection and positioning is a frequent challenge in MR‐guided TUS setups, and the method could be used to assess candidate TUS‐compatible coil configurations. It also enables the testing of sequence triggering, which is a frequent failure point in new MR‐ARFI measurement setups. As a specific example, the DC loop coil method fundamentally enabled the in vivo development of the mag‐ARFI sequence,^10^, ^21^ the primary purpose of which is to provide subtraction‐free focus visualization which is inherently registered to underlying anatomy, and hence required in vivo imaging for validation. This work would not have been possible without the DC loop coil since at the time of writing our institution had not granted IRB approval for TUS in humans.

Simulations showed that the spatial width (FWHM) of the focal phase shift produced by a DC loop coil depends on the diameter of the coil and on the distance of the imaged slice from the coil, with feasible coil diameter and slice distance pairings yielding a minimum FWHM between 10 and 20 mm. The 20 mm‐diameter coil used experimentally then produced a focal phase shift with 19 mm FWHM in phase‐ARFI and 14.4 mm FWHM in mag‐ARFI, in a slice positioned 19.2 mm from the coil. These sizes are close to but may be larger than focal sizes generated by TUS transducers. High f‐number transducers with long and wide foci are often used for TUS, and tissue mechanical response further widens the appearance of the focus in MR‐ARFI.^22^ For example, in previous non‐human primate TUS studies, a free field focus of 2.2 × 2.2 × 9.3 mm^3^ widened to 6.1 × 7.3 × 14.0 mm^3^ in high‐resolution MR‐ARFI scans,^12^ which is similar in dimension to the focus produced by our DC loop coil.

While using a DC loop coil mitigates many of the challenges associated with transcranial ultrasound for MR‐ARFI development, the method has some limitations. To produce a maximally focal phase shift, the coil's $B_{z}$ field must be aligned with $B_{0}$. For this reason, in the present study all scans were performed with the coil placed on top of the subject's head. While focal phase shifts can be produced at depths of 10–30 mm into the brain, it would be difficult to achieve deep‐brain penetration with a realistic focus size. At the same time, at any given depth, the $B_{z}$ field strength can be increased without increasing current by increasing the number of turns in the coil. The maximum number of turns possible with the DC loop coil will be limited by the logistical feasibility of securely attaching the coil to the subject's head. The benefit of more turns is achieving stronger phase shifts with lower current. Other differences to a real ultrasound transducer include the fact that no displacement signal change is produced by a transducer unless that displacement coincides with an MEG or other gradient pulse, but the DC loop coil will produce a phase shift in the image anytime between the sequence's excitation and signal readout. Another difference is the instantaneous nature of the magnetic field produced by the coil, compared to the slower evolution of tissue displacement with ultrasound. This consideration motivates starting the ultrasound pulse prior to the start of an MEG to maximize the average displacement during the MEG,^18^ which is not needed with the DC loop coil.

While this work was primarily motivated by a need for improved MR‐ARFI pulse sequences in TUS applications, the DC loop concept may also be helpful in the development of pulse sequences for other focal phase encoded methods. Alternative uses include mimicking non‐transcranial FUS and thermometry sequence development in human subjects without the need for FUS, or inducing tissue heating. This method may also be used any time a local phase shift is required, such as in mimicking flow‐induced ghosting. The work is also related work on DC shim arrays^23^ and it may be possible to achieve similar focal phase shifts using such arrays, albeit with a more sophisticated hardware setup than was required here.

## 5 CONCLUSION

DC loop coils can be used safely in humans to produce focal phase shifts that mimic those produced by transcranial ultrasound. This will facilitate the development of MR‐ARFI methods without the logistical and regulatory challenges associated with actual ultrasound application.

## CONFLICT OF INTEREST STATEMENT

The authors declare no potential conflict of interests.

## FUNDING INFORMATION

This work was supported by National Institutes of Health grants 3T32EB007509, UG3NS135551, RF1NS126144, R01MH132022. Our research group receives support from Siemens Healthineers.

## Supporting information


**Figure S1.** Alternative coil pulsing schemes. Scheme 1 (used in the experiments) triggers the pulse generator during the second MEG of an MR‐ARFI sequence in one image, and does not trigger it for the other image. Scheme 2 triggers the pulse generator prior to the refocusing pulse in one image, where the resulting phase shift is then negated by the refocusing pulse, and triggers the generator after the refocusing pulse in the other. Scheme 3 triggers the generator to generate a positive pulse in one image, and a negative pulse in the other. Unlike with FUS displacement, imaging with the DC loop can use a combination of positive and negative current through the coil to generate two phase shift images with equal and opposite phase while keeping the gradient pulsing scheme the same. Thus, coil pulsing scheme 3 works uniquely for the DC loop coil setup.
**Figure S2.** Simulated axial slices of Bz as a function of distance from the DC loop coil. The leftmost slice in the top row is 7 cm from the surface of the DC loop coil to match the geometry of the experimental setup. The slices are spaced 2 mm apart and the FOV is 44 × 44 mm^2^. A red box denotes the slice shown in Figure 2.
**Figure S3.** Axial slices of Bz as a function of distance from the DC loop coil measured in a ball phantom. The leftmost slice in the top row is 7 cm from the surface of the DC loop coil. The slices are spaced 2 mm apart and the FOV is 44 × 44 mm^2^. A red box denotes the slice shown in Figure 2.
**Figure S4.** Simulated sagittal slices of Bz as a function of distance from the center of the DC loop coil. The top left slice, and bottom right slices are furthest away from the center of the coil, and the slices are spaced 1.36 mm apart. Each slice has a FOV of 40 × 60 mm^2^. The red box denotes the slice shown in Figure 2.
**Figure S5.** Measured sagittal slices of Bz as a function of distance from the DC loop coil. The top left slice, and bottom right slices are furthest away from the center of the coil, and the slices are spaced 1.36 mm apart. Each slice has a FOV of 40 × 60 mm^2^. The red box denotes the slice shown in Figure 2.
**Figure S6.** Simulated phase shift (left) compared with measured phase shift imaged with MR‐ARFI in a ball phantom with the DC loop coil (right) at 12 mm from the surface of the coil. The maximum measured phase shift was 0.588 radians which was within 0.1 radians of agreement with the maximum simulated value of 0.526 radians.

## References

[1] Legon W , Sato TF , Opitz A , et al. Transcranial focused ultrasound modulates the activity of primary somatosensory cortex in humans. Nat Neurosci. 2014;17:322‐329. 24413698 10.1038/nn.3620

[2] Baek H , Lockwood D , Mason EJ , et al. Clinical intervention using focused ultrasound (FUS) stimulation of the brain in diverse neurological disorders. Front Neurol. 2022;13:880814. 35614924 10.3389/fneur.2022.880814 PMC9124976

[3] Lee K , Park TY , Lee W , Kim H . A review of functional neuromodulation in humans using low‐intensity transcranial focused ultrasound. Biomed Eng Lett. 2024;14:407‐438. 38645585 10.1007/s13534-024-00369-0 PMC11026350

[4] Mahoney JJ , Thompson‐Lake Daisy GY , Ranjan M , et al. Low‐intensity focused ultrasound targeting the bilateral nucleus Accumbens as a potential treatment for substance use disorder: a first‐in‐human report. Biol Psychiatry. 2023;94:e41‐e43. 37610405 10.1016/j.biopsych.2023.06.031

[5] McDannold N , Maier SE . Magnetic resonance acoustic radiation force imaging. Med Phys. 2008;35:3748‐3758. 18777934 10.1118/1.2956712 PMC2673647

[6] Phipps MA , Jonathan SV , Yang PF , et al. Considerations for ultrasound exposure during transcranial MR acoustic radiation force imaging. Sci Rep. 2019;9:16235. 31700021 10.1038/s41598-019-52443-8 PMC6838326

[7] Odéen H , Payne AH , Parker DL . Magnetic resonance acoustic radiation force imaging (MR‐ARFI). J Magn Reson Imaging. 2025;62:20–39. 39842847 10.1002/jmri.29712 PMC12179369

[8] Mohammadjavadi M , Pauly KB , Glover GH . Motion robust MR‐ARFI using timeseries single‐shot spiral acquisition. Proceedings of the 32rd International Society for Magnetic Resonance in Medicine. ISMRM; 2024:4577.

[9] Mohammadjavadi M , Ash RT , Glover GH , Pauly KB . Optimization of MR acoustic radiation force imaging (MR‐ARFI) for human transcranial focused ultrasound. Magn Reson Med. 2025;94:1060‐1071. doi:10.1002/mrm.30539 40326562 PMC12436201

[10] Sharma A , Zarcone K , Grissom WA . Magnitude preparation‐based MR‐acoustic radiation force imaging. Magn Reson Med. 2025;94:1445‐1457. 40443184 10.1002/mrm.30562 PMC12310358

[11] Bever JT , Od9en H , Todd N , Farrer AI , Parker DL . Evaluation of a three‐dimensional MR acoustic radiation force imaging pulse sequence using a novel unbalanced bipolar motion encoding gradient. Magn Reson Med. 2016;76:803‐813. 26445135 10.1002/mrm.25971 PMC5450949

[12] Luo H , Sigona MK , Manuel TJ , et al. Reduced‐field of view three‐dimensional MR acoustic radiation force imaging with a low‐rank reconstruction for targeting transcranial focused ultrasound. Magn Reson Med. 2022;88:2419‐2431. 35916311 10.1002/mrm.29403 PMC9529839

[13] Od9en H , Bever J , Hofstetter LW , Parker DL . Multiple‐point magnetic resonance acoustic radiation force imaging. Magn Reson Med. 2019;81:1104‐1117. 30257059 10.1002/mrm.27477 PMC6642829

[14] Kaye EA , Chen J , Pauly KB . Rapid MR‐ARFI method for focal spot localization during focused ultrasound therapy. Magn Reson Med. 2011;65:738‐743. 21337406 10.1002/mrm.22662 PMC4099471

[15] Holbrook AB , Ghanouni P , Santos JM , Medan Y , Pauly KB . In vivo MR acoustic radiation force imaging in the porcine liver. Med Phys. 2011;38:5081‐5089. 21978053 10.1118/1.3622610 PMC3170397

[16] Zheng Y , Marx M , Miller GW , Pauly KB . High sensitivity MR acoustic radiation force imaging using transition band balanced steady‐state free precession. Magn Reson Med. 2018;79:1532‐1537. 28631853 10.1002/mrm.26793 PMC5995567

[17] Aubry JF , Attali D , Schafer M , et al. ITRUSST consensus on biophysical safety for transcranial ultrasonic stimulation . arXiv:2311.05359 [physics] 2024. 10.1016/j.brs.2025.10.007 PMC12644226 41072763

[18] Phipps MA , Manuel TJ , Sigona MK , et al. Practical targeting errors during optically tracked transcranial focused ultrasound using MR‐ARFI and Array‐ based steering. IEEE Trans Biomed Eng. 2024;71:2740‐2748. 38640051 10.1109/TBME.2024.3391383 PMC11983265

[19] Tang KWK , Jeong J , Hsieh JC , et al. Bioadhesive hydrogel‐coupled and miniaturized ultrasound transducer system for long‐term wearable neuromodulation. Nat Commun. 2025;16:4940. 40436843 10.1038/s41467-025-60181-x PMC12119832

[20] International Electrotechnical Commission . Medical electrical equipment‐part 1: general requirements for basic safety and essential performance. International Standard IEC 60601–1, Ed. 3.1: International Electrotechnical Commission Geneva, Switzerland. International Electrotechnical Commission; 2012.

[21] Sharma A , Zarcone K , Grissom WA . Magnitude‐contrast MR‐ARFI (mag‐ARFI): subtraction‐free MR‐ARFI with inherently fused ultrasound focus and anatomy. Proceedings of the 33rd International Society for Magnetic Resonance in Medicine, Honolulu. ISMRM; 2025:0220.

[22] Payne A , De Bever J , Farrer A , et al. A simulation technique for 3D MR‐guided acoustic radiation force imaging. Med Phys. 2015;42:674‐684. 25652481 10.1118/1.4905040 PMC4297281

[23] Stockmann JP , Witzel T , Keil B , et al. A 32‐channel combined RF and B0 shim array for 3T brain imaging. Magn Reson Med. 2016;75:441‐451. 25689977 10.1002/mrm.25587 PMC4771493

