# Supplementary material for: Mimicking focused ultrasound with a loop coil in acoustic radiation force imaging
Source: Magn Reson Med. 2025 Aug 24;94(6):2529–36. doi: 10.1002/mrm.70014 (PMC12501678; doi:10.1002/mrm.70014)
Supplement: Supplementary file 1 — Figure S1. Alternative coil pulsing schemes. Scheme 1 (used in the experiments) triggers the pulse generator during the second MEG of an MR‐ARFI sequence in one image, and does not trigger it for the other image. Scheme 2 triggers the pulse generator prior to the refocusing pulse in one image, where the resulting phase shift is then negated by the refocusing pulse, and triggers the generator after the refocusing pulse in the other. Scheme 3 triggers the generator to generate a positive pulse in one image, and a negative pulse in the other. Unlike with FUS displacement, imaging with the DC loop can use a combination of positive and negative current through the coil to generate two phase shift images with equal and opposite phase while keeping the gradient pulsing scheme the same. Thus, coil pulsing scheme 3 works uniquely for the DC loop coil setup. Figure S2. Simulated axial slices of Bz as a function of distance from the DC loop coil. The leftmost slice in the top row is 7 cm from the surface of the DC loop coil to match the geometry of the experimental setup. The slices are spaced 2 mm apart and the FOV is 44 × 44 mm2. A red box denotes the slice shown in Figure 2. Figure S3. Axial slices of Bz as a function of distance from the DC loop coil measured in a ball phantom. The leftmost slice in the top row is 7 cm from the surface of the DC loop coil. The slices are spaced 2 mm apart and the FOV is 44 × 44 mm2. A red box denotes the slice shown in Figure 2. Figure S4. Simulated sagittal slices of Bz as a function of distance from the center of the DC loop coil. The top left slice, and bottom right slices are furthest away from the center of the coil, and the slices are spaced 1.36 mm apart. Each slice has a FOV of 40 × 60 mm2. The red box denotes the slice shown in Figure 2. Figure S5. Measured sagittal slices of Bz as a function of distance from the DC loop coil. The top left slice, and bottom right slices are furthest away from the center of the coil, and the slices [file MRM-94-2529-s001.doc]

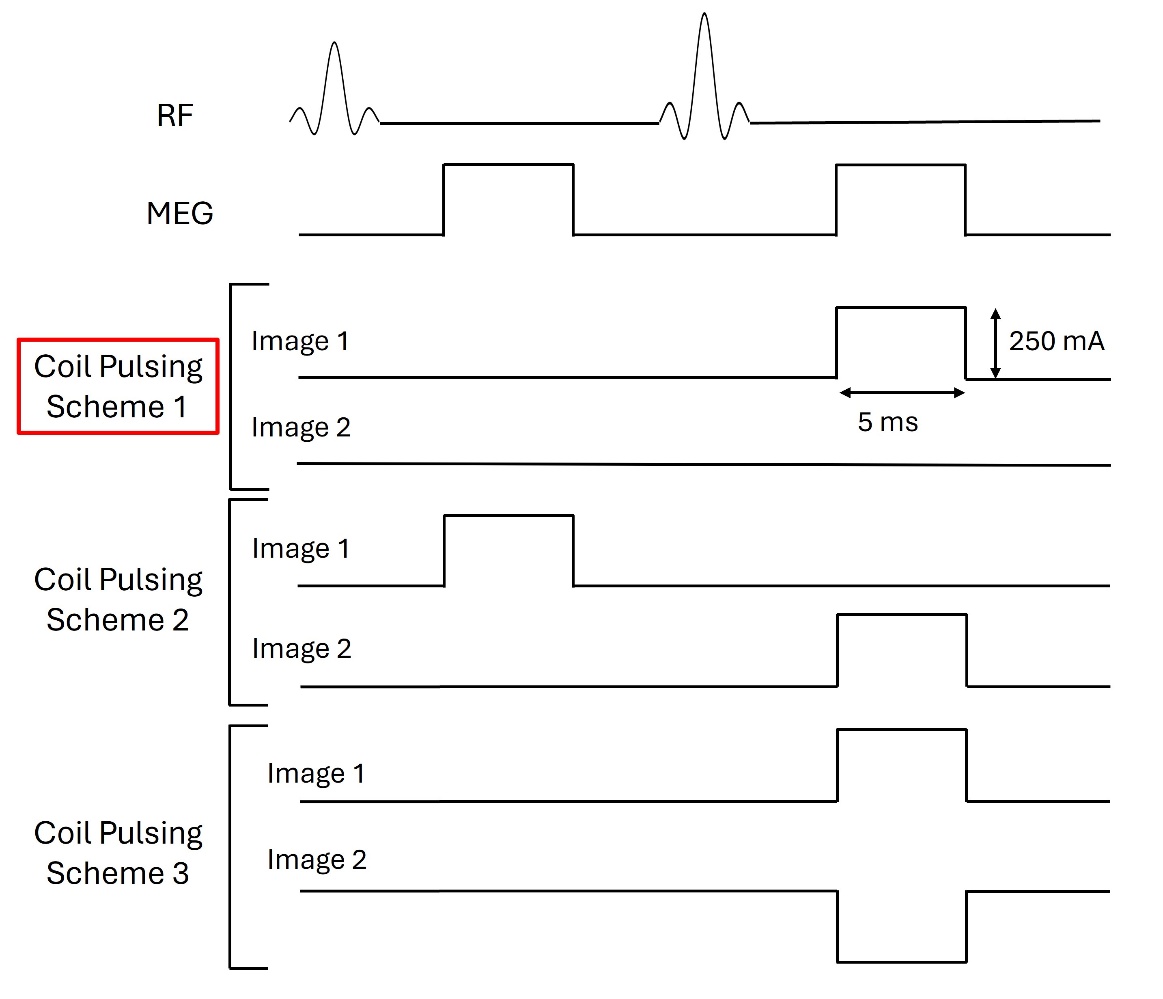
Pulsing Schemes

Supporting Information Figure 1:

Alternative coil pulsing schemes are shown. Scheme 2 triggers the pulse generator prior to the refocusing pulse in one image, where the resulting phase shift is then negated by the refocusing pulse, and triggers the generator after the refocusing pulse in the other. Scheme 3 triggers the generator to generate a positive pulse in one image, and a negative pulse in the other.

Unlike with FUS displacement, we can use a combination of positive and negative current through the coil to generate two phase shift images with equal and opposite phase while keeping the gradient pulsing scheme the same. Thus, coil pulsing scheme 3 works uniquely for the DC loop coil setup.

Simulated Bz map axial


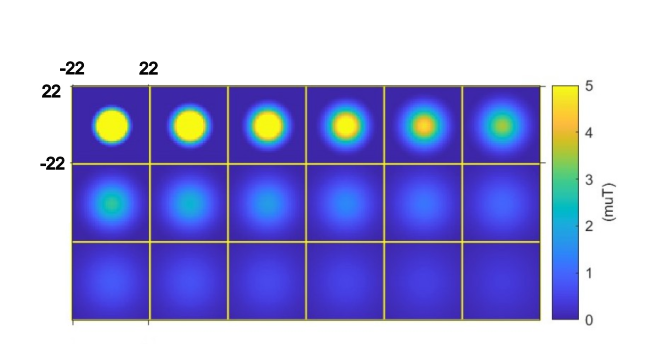

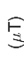


7 mm

41 mm

Supporting Information Figure 2:

Simulated axial slices of Bz as a function of distance from the DC loop coil. The leftmost slice in the top row is assumed to be at approximately 7 cm from the surface of the DC loop coil to match the geometry of the experimental setup. Each slice represents 2 mm and has a FOV of 44 x 44 mm^2. A red box denotes the slice shown in Figure 2 of the paper.

Measured Bz map axial


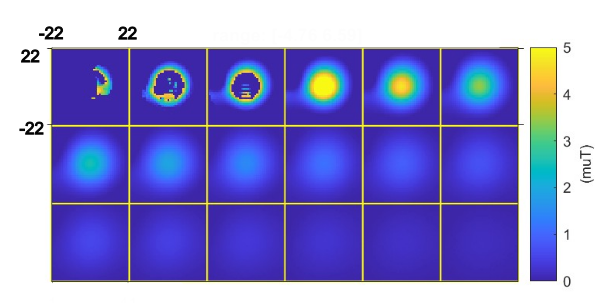

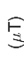


41 mm

7 mm

Supporting Information Figure 3:

Axial slices of Bz as a function of distance from the DC loop coil measured in a ball phantom. The leftmost slice in the top row is assumed to be at a distance of 7 cm from the surface of the DC loop coil due to the outer shell of the ball phantom and coil placement. Each slice represents 2 mm and has a FOV of 44 x 44 mm^2. A red box denotes the slice shown in Figure 2 of the paper.

Simulated Bz Sagittal

Supporting Information Figure 4:


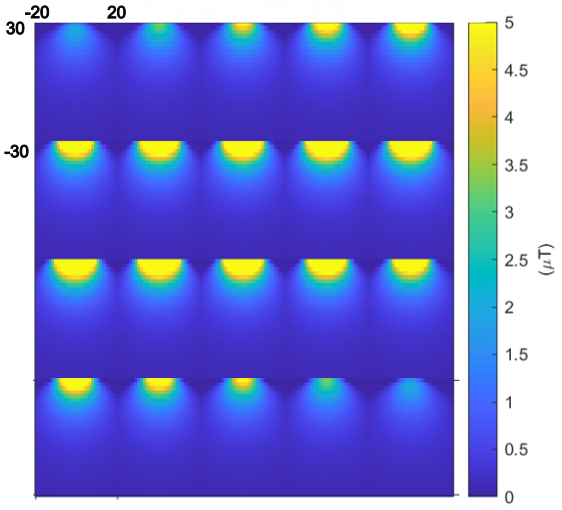


-14 mm

14 mm

Simulated sagittal slices of Bz as a function of distance from the center of the DC loop coil. The top left slice, and bottom right slices are furthest away from the center of the coil, and each slice is spaced 1.36 mm away from one another. Each slice has a FOV of 40 x 60 mm^2. A red box denotes the slice shown in Figure 2 of the paper.

Measured Bz map sagittal


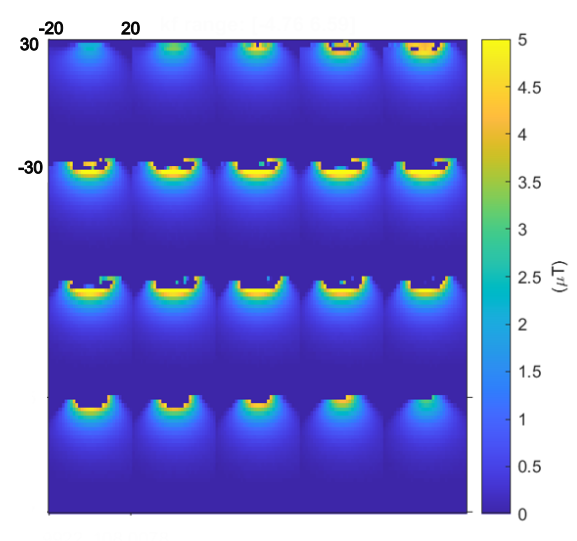


14 mm

-14 mm

Supporting Information Figure 5:

Measured sagittal slices of Bz as a function of distance from the DC loop coil. The top left slice, and bottom right slices are furthest away from the center of the coil, and each slice is spaced 1.36 mm away from one another. Each slice has a FOV of 40 x 60 mm^2. A red box denotes the slice shown in Figure 2 of the paper.


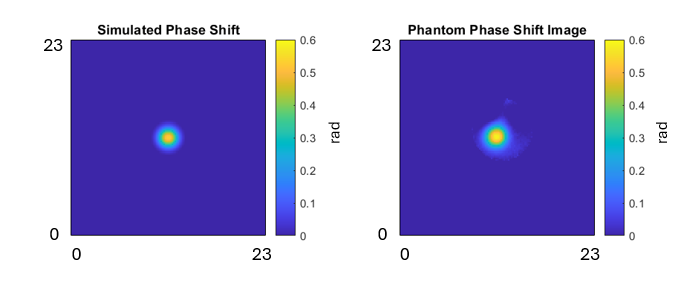


Supporting Information Figure 6:

Simulated phase shift (left) compared with measured phase shift imaged with MR-ARFI in a ball phantom with the DC loop coil (right) at 12 mm from the surface of the coil. The maximum measured phase shift was 0.588 radians which was within 0.1 radians of agreement with the maximum simulated value of 0.526 radians.
